# Two Splicing Variants of *OsNPF7.7* Regulate Shoot Branching and Nitrogen Utilization Efficiency in Rice

**DOI:** 10.3389/fpls.2018.00300

**Published:** 2018-03-08

**Authors:** Weiting Huang, Genxiang Bai, Jie Wang, Wei Zhu, Qisen Zeng, Kai Lu, Shiyong Sun, Zhongming Fang

**Affiliations:** ^1^Center of Applied Biotechnology, Wuhan Institute of Bioengineering, Wuhan, China; ^2^National Key Laboratory of Crop Genetic Improvement, Huazhong Agricultural University, Wuhan, China

**Keywords:** rice, nitrogen, transporter, tiller number, grain number

## Abstract

Rice includes 93 nitrate and peptide transporters family (NPF) members that facilitate the soil uptake and internal reallocation of nitrogen for growth and development. This study demonstrated that *OsNPF7.7* had two splicing variants, and altered expression of each variant could regulate shoot branching and nitrogen utilization efficiency (NUtE) in rice. The expression of both variants was down-regulated in the buds by increased nitrogen level in the *Japonica* rice variety ZH11. The expression level of long-variant *OsNPF7.7-1* was higher in panicles at reproductive stage, however, the expression level of short-variant *OsNPF7.7-2* was higher in buds and leaves at vegetative stage compared to each other in ZH11. OsNPF7.7-1 was localized in the plasma membrane, whereas OsNPF7.7-2 was localized in the vacuole membrane. Furthermore, the results indicated that the expression level of each variant for *OsNPF7.7* determined axillary bud outgrowth, and then influenced the rice tiller number. Overexpression of *OsNPF7.7-1* could promote nitrate influx and concentration in root, whereas overexpression of *OsNPF7.7-2* could improve ammonium influx and concentration in root. RNAi and *osnpf7.7* lines of *OsNPF7.7* showed an increased amount of amino acids in leaf sheaths, but a decreased amount in leaf blades, which affected nitrogen allocation and plant growth. The elevated expression of each variant for *OsNPF7.7* in ZH11 enhanced NUtE using certain fertilization regimes under paddy field conditions. Moreover, overexpression of each variant for *OsNPF7.7* in KY131 increased significantly the filled grain number per plant. Thus, increased each variant of *OsNPF7.7* has the potential to improve grain yield and NUtE in rice.

## Introduction

The application of nitrogen is one possible measure to obtain higher yield, and China applies more nitrogen fertilizers than any other country in the world (Narits, 2010). The loss of nitrogen is not only a serious waste of limited nutrient resources, but also negatively affects the environment through water and air emissions, eutrophication, ground water pollution, and soil acidification (McAllister et al., 2012). A potential solution to this issue could be the increase of nitrogen use efficiency (NUE) in crop plants, including rice (Xu et al., 2012). NUE is divided into two components: N uptake efficiency (NUpE) and Nitrogen utilization efficiency (NutE) (Han et al., 2015). Therefore, NUE is a crucial strategy to increase the grain yield, which is mainly controlled by tiller number per plant, grain number per panicle, and thousand-grain weight in rice (Li et al., 2003; Liang et al., 2014).

Rice tiller experience two distinct stages in its development: the formation of an axillary bud at each leaf axil and the outgrowth of the axillary bud (Li et al., 2003). Nitrogen could enhance the metabolism of carbon and nitrogen, and the synthesis of endogenous hormones such as cytokinin, and then promote the elongation of axillary bud and the production of tiller (Sakakibara et al., 2006; Ohashi et al., 2017). Plants have evolved multiple transport systems to facilitate nitrogen uptake from the soil and internal reallocation involving in growth and development including rice tiller number (Rentsch et al., 2007; Tsay et al., 2007; Hu et al., 2015). Among Rice 93 nitrate and peptide transporters family (NPF) transporters (NPF: NRT1, low-affinity nitrate transporter; PTR, di/tripeptide transporter) (Zhao et al., 2010; Léran et al., 2014), only nine members have been functionally studied. OsNRT1 (OsNPF8.9) is a low-affinity nitrate transporter (Lin et al., 2000). *SP1* (*OsNPF4.1*) determines the rice panicle size (Li et al., 2009). OsPTR6 (OsNPF7.3) transports di/tripeptides (Ouyang et al., 2010) and improves rice growth (Fan et al., 2014). Overexpression of the splicing gene *OsPTR9* (*OsNPF8.20-2*) enhances tiller number and NUtE in rice (Fang et al., 2013).

The rice nitrate transporter OsNPF2.4 functions in low-affinity acquisition and long-distance transport (Xia et al., 2015). *OsNRT1.1B* (*OsNPF6.5*) *Indica* variant enhances nitrate uptake, tiller number and NUtE in rice (Hu et al., 2015). Disruption of the rice nitrate transporter *OsNPF2.2* inhibits the root-to-shoot nitrate transport and impedes vascular development (Li et al., 2015). Knockdown of low-affinity nitrate transporter *OsNPF7.2* retards rice growth under high nitrate supply (Hu et al., 2016). OsPTR7 (NPF8.1-1) is involved in dimethyl arsenate accumulation in rice grains (Tang et al., 2017). Furthermore, *OsNPF7.3* is induced by organic nitrogen, and contributes to tiller number and grain yield (Fang et al., 2017).

Among the important genes identified in previous studies, we identified *OsNRT1* (*OsNPF8.9-1*, Lin et al., 2000), *OsPTR9* (*OsNPF8.20-2*, Fang et al., 2013), *OsNPF2.4-1* (Xia et al., 2015), and *OsPTR7* (*OsNPF8.1-1*, Tang et al., 2017) as alternative splicing genes in the *OsNPF* family by searching the Rice Genome Sequence Annotation^1^. As we know, alternative splicing is a pattern to increase proteins and enrich the function of a gene (Graveley, 2001). So we speculated that the NPF gene with splicing variants may play an important role in nitrogen translocation and rice growth. In this study, we determined that altered expression of both splicing variants of *OsNPF7.7* (*OsPTR10*) in the NPF family could regulate shoot branching and NUtE in rice. The results could provide diverse alternatives for splicing formation and regulation with respect to function research and NUtE of *OsNPF* splicing members in rice.

## Materials and Methods

### The Acquisition of Transgenic Rice With Altered Expression of *OsNPF7.7*

A cDNA 1,710 bp fragment of *OsNPF7.7-1* or a cDNA 1,428 bp fragment of *OsNPF7.7-2*, containing the open reading frame (ORF) of each *OsNPF7.7* variant, was inserted downstream of the *35S* promoter in pCAM1301 with *Bgl*II and *Afl*II, respectively, producing *OsNPF7.7-1* or *OsNPF7.7-2* overexpression plasmid. To generate *OsNPF7.7* RNAi construct, two 263 bp cDNA fragments in common sequence of both variants for *OsNPF7.7* were amplified by PCR and inserted downstream of the *Ubi-1* promoter in rice RNAi vector pTCK303 (Wang et al., 2004) with *BamH*I/*Kpn*I and *Spe*I/*Sac*I, respectively. The homozygous T-DNA insertion mutant *osnpf7.7* in ZH11 background was obtained from the Rice Mutant Database of Huazhong Agricultural University (Mutant ID: 04Z11EH19, T-DNA was inserted at site 1091 bp below the first ATG in *OsNPF7.7*^2^). To generate *OsNPF7.7*-GFP, *OsNPF7.7-1* or *OsNPF7.7-2* ORF (lacking the stop codon) was amplified by PCR and was cloned in front of the green fluorescent protein (GFP) coding region in pCAM1302 vector^3^ with *Bgl*II and *Spe*I, respectively. *Agrobacterium* strain *EHA*105 mediated vectors were further transformed into the *Japonica* rice varieties ZH11 or KY131 (Hiei et al., 1997). The T2 or T3 transgenic lines were used for PCR detection. All the primers were listed in Supplementary Table S1.

### Subcellular Localization of OsNPF7.7

To determine the subcellular localization of both variants of OsNPF7.7, p35S-*OsNPF7.7-1*-*GFP*, p35S-*OsNPF7.7-2*-*GFP*, and p35S-*GFP* were transformed into rice protoplasts as described previously (Li et al., 2015). Protoplasts were acquired from leaf sheaths of rice seedlings after sowing for 7–15 days. The co-expressed markers were plasma membrane protein OsMCA1 fusing with mCherry (Kurusu et al., 2012) and vacuole membrane protein AtTPK fusing with mkate (Voelker et al., 2006). The fluorescence was observed using a confocal laser scanning microscope (Leica SP8 AOBS, Wetzlar, Germany).

### Rice Growth Under Greenhouse Conditions

Germinated seeds were cultured at 28°C under white light with a 16 h light/8 h dark photoperiod for 14 days by basic rice culture solution (Yoshida et al., 1976). The composition of the basic rice solution was as follows: 1.0 mM NH_4_NO_3_, 0.32 mM NaH_2_PO_4_, 0.51 mM K_2_SO_4_, 1 mM CaCl_2_, 1.65 mM MgSO_4_, 8.9 μM MnSO_4_, 0.5 μM Na_2_MoO_4_, 18.4 μM H_3_BO_3_, 0.14 μM ZnSO_4_, 0.16 μM CuSO_4_, and 40 μM FeSO_4_. The nitrogen content was adjusted in each experiment. Hydroponic experiments were conducted in the hydroponic culture box with size 525 mm × 360 mm × 230 mm covering with cystosepiment by the basic rice culture solution under greenhouse condition. To analyse *OsNPF7.7-1* and *OsNPF7.7-2* expression in the presence of different nitrogen sources, seedlings of ZH11 variety were grown in the basic rice culture solution supplemented with one of the following as a sole nitrogen source: 0.5 mM NaNO_3_, 2.0 mM NaNO_3_, 5.0 mM NaNO_3_, 0.25 mM (NH_4_)_2_SO_4_, 1.0 mM (NH_4_)_2_SO_4_, 2.5 mM (NH_4_)_2_SO_4_, 0.25 mM NH_4_NO_3_, 1.0 mM NH_4_NO_3_, or 2.5 mM NH_4_NO_3_. The nutrient solution was renewed every 3 days. Samples were harvested for RNA extraction after 3 weeks. To analyze *OsNPF7.7-1* and *OsNPF7.7-2* expression in the different tissues of rice, seedlings ZH11 were grown in hydroponic culture box with size 525 mm × 360 mm × 230 mm by the soil under greenhouse condition continuously. Samples were harvested for RNA extraction at vegetative stage and reproductive stage. The greenhouse condition is 32°C for sodium lamp 400 w 14 h in the daytime and 25°C for dark 10 h in the evening.

### Rice Growth Under Paddy Field Conditions

The rice grew in paddy at season June to October from year 2014 to 2017 at rice experimental field of Huazhong Agricultural University. The 1/3 of the total amount of fertilizer (N/P_2_O_5_/K_2_O = 19%/7%/14%; Hubei Batewang Chemical Co., Ltd.) was applied before seedling transplanting, 1/3 at tillering stage and 1/3 at heading stage. The initial nitrogen level of soil paddy was 30 kg/hm^2^. Generally, the number of rice plants was 30 for each experiment and the planting density was 19.98 cm × 19.98 cm. For the field yield trials, the number of rice plants was 100 for each transgenic plants and ZH11. For paddy field test of fertilization regimes, the amount of NH_4_NO_3_ treatment was 0, 90, 180, and 270 kg/hm^2^. For each treatment, nitrogen was fractionated: 1/3 of the total amount at seedling transplanting, 1/3 at tillering stage and 1/3 at heading stage. Phosphorus and potassium provided as KH_2_PO_4_ and KCl, respectively, were applied before transplanting at the amount of 180 kg/hm^2^ for all treatments.

### Nitrogen Influx, Concentration, and Utilization Efficiency Analysis

To analysis the nitrate and ammonium influx and concentration, 2 week seedlings of ZH11, and transgenic plants of *OsNPF7.7* were placed in basic rice culture solution without nitrogen for 3 days. The nitrogen-starved seedlings were transferred to culture solution containing 2.0 mM NaNO_3_ or 1.0 mM (NH_4_)_2_SO_4_ for 24 h. NO_3_^-^ influx was calculated as the difference in NO_3_^-^ concentration between the 2.0 mM nitrate-treatment and nitrate-starved plants in an hour. NO_3_^-^ concentration was determined by the colorimetric method (Cai et al., 2009). Ammonium influx was calculated as the difference in NH_4_^+^ concentration between the 2.0 mM ammonium-treatment and ammonium-starved plants in an hour. Ammonium concentration was measured by the colorimetric method (Kemsley et al., 2001). Total free amino acid concentration was measured by the ninhydrin method (Fang et al., 2013). Sole free amino acid concentration was measured by HPLC method using amino acid analyzer L-8800 HITACHI. Total nitrogen content and total protein content were determined using the semi-micro Kjeldahl method by using a nitrogen analyzer (Smart Chem 200, Westco, Italy). NUtE was determined as grain dry weight (g)/[total N in grain (g) + total N in straw (g)].

### RNA Extraction and qRT-PCR

Total RNA was extracted using TRIzol reagent (Invitrogen, Beijing, China). First-strand cDNA was synthesized using oligo (dT) primers and MLV reverse transcriptase (TaKaRa Bio, Beijing, China). qRT-PCR was carried out using SYBR Green Premix (TaKaRa Bio) and monitored with the 7500 qRT-PCR system (Applied Biosystems, Foster City, CA, United States). To detection the expression level of two variants for *OsNPF7.7*, the primers sites for longer variant *OsNPF7.7-1* were situated at the sequence of variant *OsNPF7.7-1* its own, and the primers of *OsNPF7.7-2* were designed at the common sequence of both two variants. Next, the actual expression amount of variant *OsNPF7.7-2* was that both variants amount of *OsNPF7.7* deduced the amount of *OsNPF7.7-1*. The primers for qRT-PCR were listed in Supplementary Table S1.

### Bud Outgrowth Analysis

To analyze bud outgrowth of different transgenic plants of *OsNPF7.7*, 2 week seedlings were grown in hydroponic culture box with size 525 mm × 360 mm × 230 mm in the rice culture solution under greenhouse condition. The greenhouse condition is 32°C for sodium lamp 400 w 14 h in the daytime and 25°C for dark 10 h in the evening. The nutrient solution was renewed every 3 days. The length of bud outgrowth was measured using a stereo microscope with ImageJ software.

### Statistical Analysis

For all treatments, the statistical differences were indicated by asterisks, and the student’s *t*-test allowing the determination of the significance between two sets of data was performed using the SPSS 10 software (IBM, Inc.). Significant levels: ^∗∗∗^*p* < 0.001, ^∗∗^*p* < 0.01, ^∗^*p* < 0.05; ^NS^*p* > 0.05.

## Results

### Two Splicing Variants of *OsNPF7.7* Have Different Expression Patterns

**FIGURE 1:**
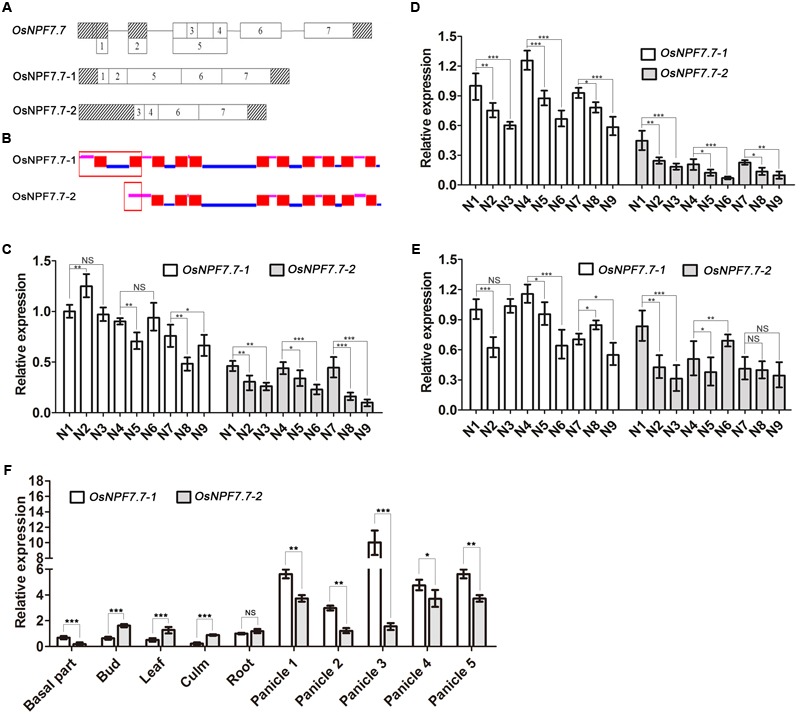
Two splicing variants of *OsNPF7.7* have different expression patterns. Gene structure **(A)** and transmembrane domains **(B)** of two splicing variants for *OsNPF7.7*. Root **(C)**, axillary bud **(D)**, and leaf **(E)** expression patterns of splicing variants for *OsNPF7.7* under different nitrogen conditions. **(F)** The expression pattern of OsNPF7.7-1 and OsNPF7.7-2 variants in different rice tissues. The pink lines represent extracellular membrane domains in **(B)**, and the blue lines represent intracellular membrane domains in **(B)**. The solid red boxes represent transmembrane domains in **(B)**, and the hollow red box represents specific domain of *OsNPF7.7-1* in (B). Panicle 1 represents early booting stage panicle in **(F)**, panicle 2 represents late booting stage panicle in **(F)**, panicle 3 represents early heading stage panicle in **(F)**, panicle 4 represents late heading stage panicle in **(F)**, and panicle 5 represents filling stage panicle in **(F)**. Seedlings of ZH11 for different nitrogen treatments **(C–E)** were cultured 3 weeks under greenhouse condition in the basic rice culture solution containing one of the following as a sole nitrogen source: 0.5 mM KNO_3_ (N1), 2.0 mM KNO_3_ (N2), 5.0 mM KNO_3_ (N3), 0.25 mM (NH_4_)_2_SO_4_ (N4), 1 mM (NH_4_)_2_SO_4_ (N5), 2.5 mM (NH_4_)_2_SO_4_ (N6), 0.25 mM NH_4_NO_3_ (N7), 1 mM NH_4_NO_3_ (N8), or 2.5 mM NH_4_NO_3_ (N9). Seedlings of ZH11 for expression pattern in different rice tissues **(F)** were cultured under greenhouse condition in the soil pots. All samples were collected for RNA extraction. Three replicates have been used for each experiment. Significant levels (N2 or N3 VS. N1; N5 or N6 VS. N4; N8 or N9 VS. N7; Variant *OsNPF7.7-1* VS. Variant *OsNPF7.7-2*): ^∗∗∗^*p* < 0.001, ^∗∗^*p* < 0.01, ^∗^*p* < 0.05; ^NS^*p* > 0.05. Error bars depict the SD (*n* = 3).

We found that *OsNPF7.7* in the *OsNPF* family has two splicing variants (the long-splicing variant *OsNPF7.7-1* and the short-splicing variant *OsNPF7.7-2*) with different exons (**Figure 1A**). The splicing proteins were localized at the membrane and contained 11 transmembrane helices in the long variant and nine in the short variant (**Figure 1B**). To gain insight into the comprehensive roles of the alternative splicing mRNA forms of *OsNPF7.7* in response to various forms of nitrogen in rice, their expression patterns were investigated by qRT-PCR using inorganic nitrogen (NO_3_^-^, NH_4_^+^, or NH_4_NO_3_) as a sole nitrogen source. Higher levels of long splicing mRNA existed in *OsNPF7.7*, indicating that different mRNA splicing forms of a gene were expressed selectively in response to the form of nitrogen. In response to all nitrogen treatments, the expression of *OsNPF7.7-2* was downregulated in the roots (**Figure 1C**) and axillary buds (**Figure 1D**) from low to high nitrogen concentration, whereas the expression of *OsNPF7.7-1* was downregulated only in the axillary buds (**Figures 1C–E**). To further determine the expression level of two splicing variants for *OsNPF7.7* in rice, qRT-PCR experiment of each variant for *OsNPF7.7* was measured with different rice tissues (**Figure 1F**). The result showed that the expression of *OsNPF7.7-1* is higher in panicles at reproductive stage, however, the expression of *OsNPF7.7-2* is higher in buds and leaves at vegetative stage compared to each other (**Figure 1F**).

### Subcellular Localization of OsNPF7.7

**FIGURE 2:**
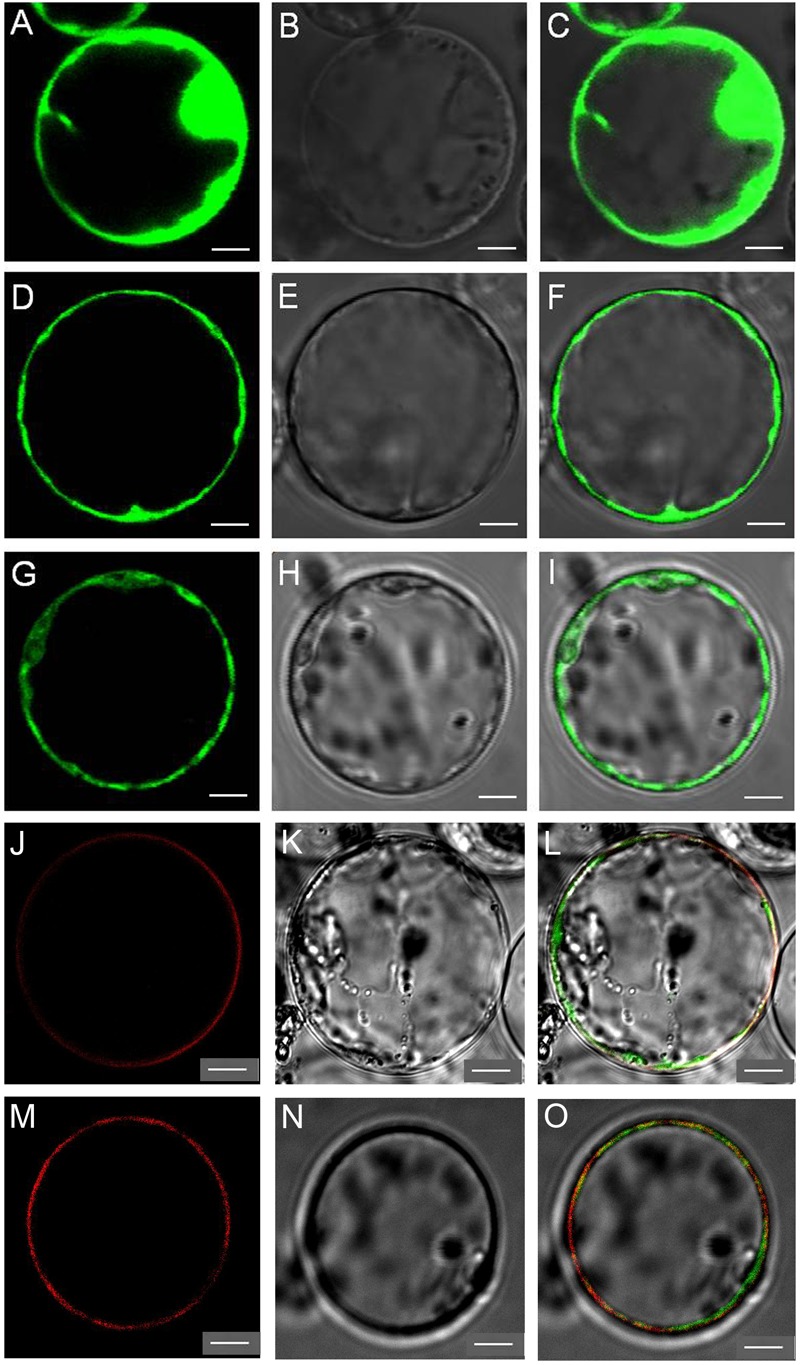
Two splicing variants of OsNPF7.7 are localized at different membranes. **(A–C)** Free GFP expression in rice protoplasts. **(D–F)** OsNPF7.7-1-GFP expression in rice protoplasts. **(G–I)** OsNPF7.7-2-GFP expression in rice protoplasts. **(J–L)** OsNPF7.7-1-GFP co-expression with the plasma membrane protein OsMCA1 fusing with mCherry in rice protoplasts. **(M–O)** OsNPF7.7-2-GFP co-expression with vacuolar membrane protein AtTPK fusing with mkate in rice protoplasts. Fluorescence images of GFP **(A,D,G)**, mcherry **(J)**, and mkate **(M)**, corresponding bright-field images **(B,E,H,K,N)**, and merged images **(C,F,I,L,O)**. Three replicates have been used for each experiment. Bars = 5 μm **(A–L)**.

To determine the intracellular localization of OsNPF7.7, we found that the 35S: GFP (control) produced green fluorescence in the plasma membrane and the nucleus (**Figures 2A–C**), but the transient expression of the long variant OsNPF7.7-1-GFP in rice protoplasts produced green fluorescence in the plasma membrane (**Figures 2D–F**), whereas that of the short variant OsNPF7.7-2-GFP produced green fluorescence in the vacuole membrane (**Figures 2G–I**). Furthermore, the results showed that the OsNPF7.7-1-GFP fusion protein signal was localized at plasma membrane (**Figures 2J–L**) with overlap signal of the plasma membrane marker OsMCA1 fusing with mCherry, and OsNPF7.7-2-GFP fusion protein signal was localized at vacuolar membrane (**Figures 2M–O**) with overlap signal of vacuolar membrane marker AtTPK fusing with mkate. Overall, these data indicated that two variants of OsNPF7.7 had different localization patterns.

### Altered Expression of Both Variants of *OsNPF7.7* Controls Tiller Number Through Regulation of Axillary Bud Outgrowth

**FIGURE 3:**
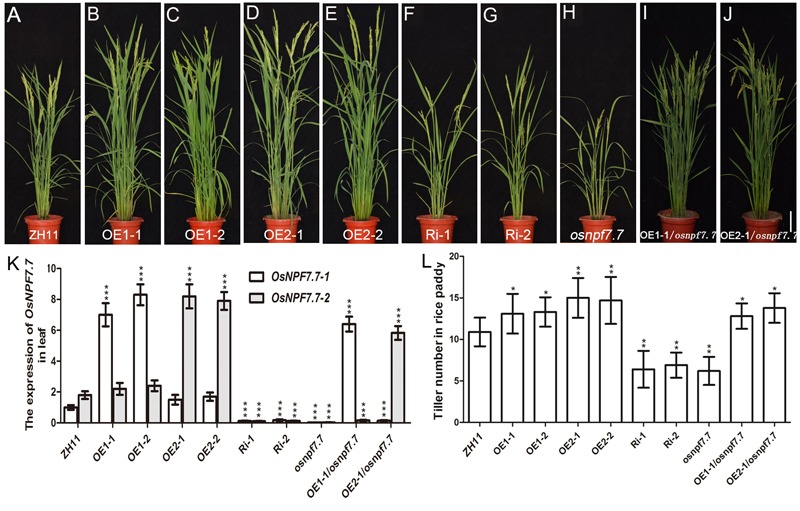
Phenotype of paddy field-grown *OsNPF7.7* transgenic plants in ZH11. Phenotype of paddy field-grown wild-type plants ZH11 **(A)** and *OsNPF7.7-1*-overexpressing in ZH11 background lines (OE1-1 and OE1-2; **B,C**), *OsNPF7.7-2*-overexpressing in ZH11 background lines (OE2-1 and OE2-2; **D,E**), *OsNPF7.7*-RNAi lines (Ri-1 and Ri-2; **F,G**), *osnpf7.7* **(H)**, *OsNPF7.7-1*-overexpressing in *osnpf7.7* background line **(I)**, and *OsNPF7.7-2*-overexpressing in *osnpf7.7* background line **(J)**. Mutant *osnpf7.7* was crossed with each overexpression lines OE1-1 or OE2-1, and F2 plants were used in experiments. Expression of *OsNPF7.7-1* and *OsNPF7.7-2* in the leaves **(K)** and the tiller number **(L)** were measured. Three replicates have been used for each experiment. Significant levels (each transgenic line VS. ZH11): ^∗∗∗^*p* < 0.001, ^∗∗^*p* < 0.01, ^∗^*p* < 0.05. Error bars depict the SD (*n* = 30). Bar = 15 cm **(A–J)**.

To determine the function of *OsNPF7.7* in rice plants, we generated *OsNPF7.7* overexpressing and RNAi transgenic rice plants (**Figure 3**). The tiller number of *OsNPF7.7-1* overexpressing lines (OE1-1, OE1-2; **Figures 3B,C,K,L**) and *OsNPF7.7-2* overexpressing lines (OE2-1, OE2-2; **Figures 3D,E,K,L**) was superior to that of ZH11 (**Figures 3A,K,L**). RNAi lines (common sequence RNAi of two variants for *OsNPF7.7*) showed a relatively low tiller number, dwarfism, and short panicles (**Figures 3F,G,K,L**). Therefore, the tiller number of mutant *osnpf7.7* (common sequence knockout of two variants for *OsNPF7.7*) was significantly lower than that of ZH11 (**Figures 3H,K,L**), but the tiller number was majorly recovered when either variant of *OsNPF7.7* was overexpressed in *osnpf7.7* (**Figures 3I–L**).

**FIGURE 4:**
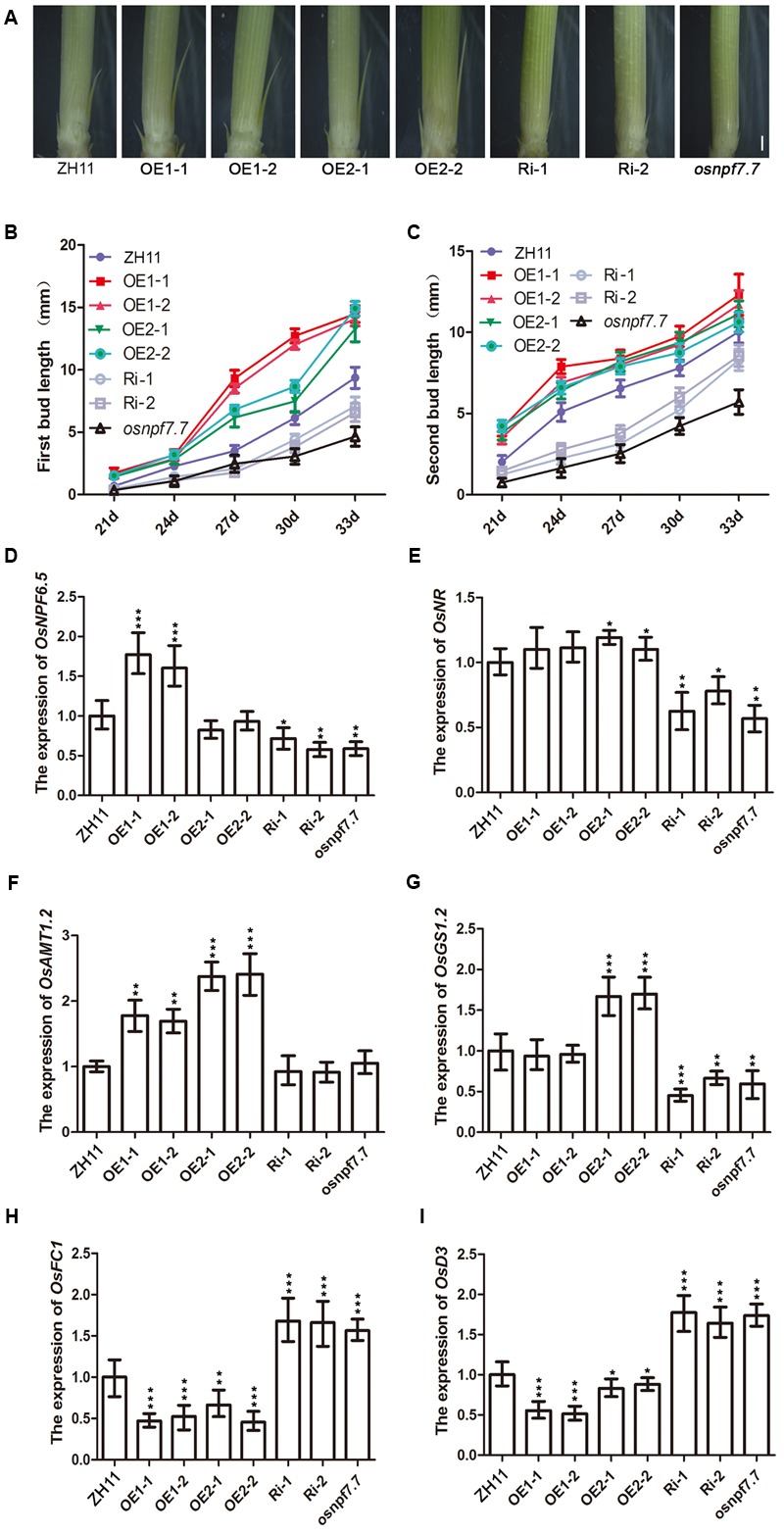
Phenotype of axillary bud outgrowth and expression of related gene of *OsNPF7.7* transgenic plants in ZH11. **(A)** Axillary buds were cultured in the basic rice culture solution containing 1 mM NH_4_NO_3_ under greenhouse condition at 27 days after sowing. First bud length **(B)** and second bud length **(C)** was measured at 21–33 days after sowing. Relative expression levels of *OsNPF6.5* **(D)**, *OsNR* **(E)**, *OsAMT1.2* **(F)**, and *OsGS1.2* **(G)** was measured in the root of transgenic plants at 27 days after sowing. Relative expression level of *OsFC1* **(H)** and *OsD3* **(I)** was measured in the axillary bud of transgenic plants at 27 days after sowing. Three replicates have been used for each experiment. Significant levels (each transgenic line VS. ZH11): ^∗∗∗^*p* < 0.001, ^∗∗^*p* < 0.01, ^∗^*p* < 0.05. Error bars depict the SD (*n* = 3). Bar = 1 mm **(A)**.

To further examine whether *OsNPF7.7* regulated the axillary bud formation or axillary bud elongation and controlled the rice tiller number, we tested the responses of *OsNPF7.7* transgenic plants raised in hydroponic culture. The results showed that the length of first (**Figures 4A,B**) and second (**Figures 4A,C**) axillary buds of OE1 and OE2 plants were longer at the seedling stage than those of ZH11, but the length of buds was shorter in RNAi lines and *osnpf7.7* plants than that in ZH11 (**Figures 4A–C**). Furthermore, the expression of *OsNPF6.5* (**Figure 4D**) and *OsAMT1.2* (**Figure 4F**) increased in OE1 lines; the expression of *OsNR* (**Figure 4E**), *OsAMT1.2* (**Figure 4F**) and *OsGS1.2* (**Figure 4G**) increased in OE2 lines; and the expression of *OsNPF6.5* (**Figure 4D**), *OsNR* (**Figure 4E**), *OsAMT1.2* (**Figure 4F**), and *OsGS1.2* (**Figure 4G**) decreased significantly in Ri and *osnpf7.7* plants. Furthermore, we indicated that overexpression of *OsNPF7.7-1* promoted the nitrate influx (Supplementary Figure S1A) and nitrate concentration in rice (Supplementary Figure S1B), whereas overexpression of *OsNPF7.7-2* enhanced significantly the ammonium influx (Supplementary Figure S1C) and concentration in rice (Supplementary Figure S1D). The expression of the outgrowth bud elongation marker *OsFC1* dramatically decreased in OE1 and OE2 lines, but increased in RNAi and *osnpf7.7* plants (**Figure 4H**). In addition, the strigolactone signaling gene *OsD3* also decreased in OE1 and OE2 lines, but increased in RNAi and *osnpf7.7* plants (**Figure 4I**).

### Effects of Two Splicing Variants of *OsNPF7.7* on Nitrogen Translocation to the Leaves and Recycling to the Panicle

**FIGURE 5:**
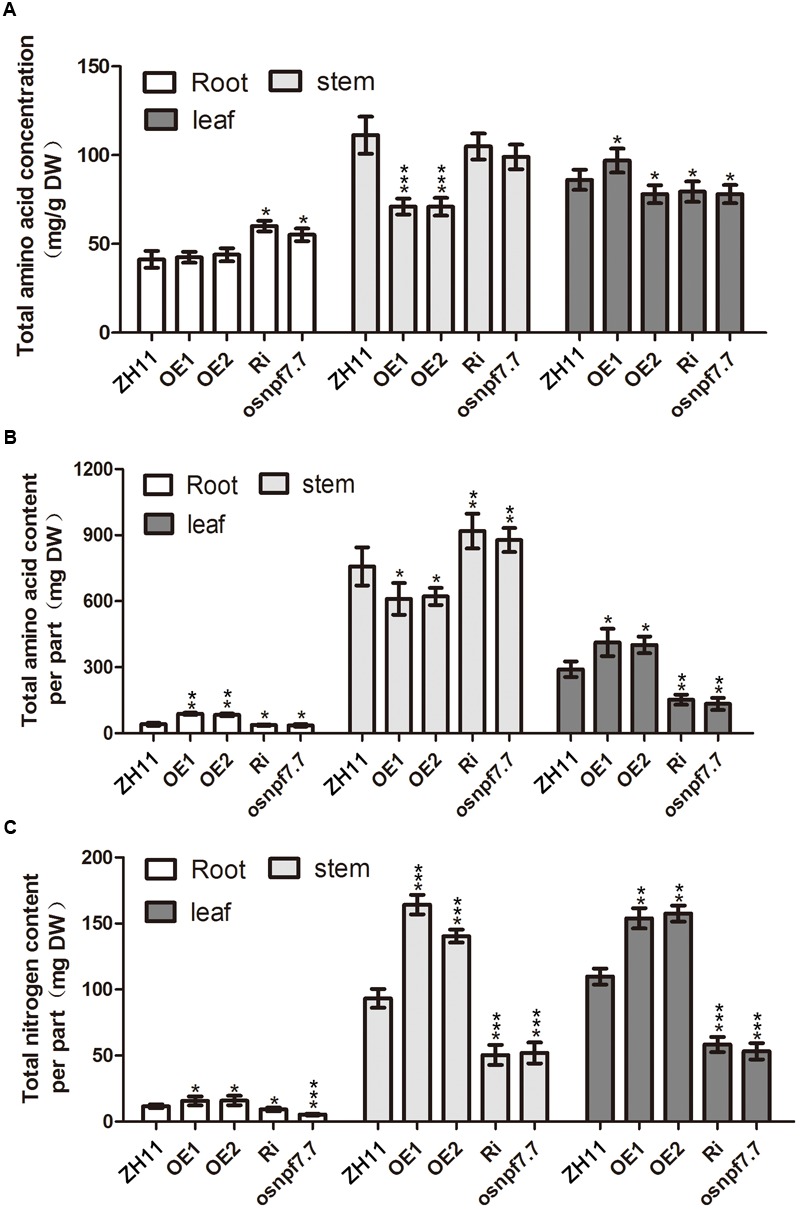
Amino acid and total nitrogen content in ZH11 transgenic plants. Total amino acid concentration **(A)**, total amino acid content per part **(B)**, and total nitrogen content per part **(C)** of 3-month-old cultured ZH11 and transgenic seedlings using basic rice culture solution supplemented with 1.0 mM NH_4_NO_3_ as a nitrogen source. OE1, OE2, and Ri indicated that mixed equal-amount which extracted from each three OE1, OE2, and Ri lines, respectively. Three replicates have been used for each experiment. Significant levels (each transgenic line VS. ZH11): ^∗∗∗^*p* < 0.001, ^∗∗^*p* < 0.01, ^∗^*p* < 0.05. Error bars depict the SD (*n* = 3).

To investigate the reasons that *OsNPF7.7* affects the rice tiller number, we measured the free amino acid and total nitrogen content in the roots, stems, and leaves of OE, Ri, and *osnpf7.7* lines (**Figure 5**). The results showed that the free amino acid content was higher in the roots of Ri and *osnpf7.7* plants than in those of ZH11, but lower in the leaves (**Figure 5A**). The amino acids that mainly accumulated in the root of Ri and *osnpf7.7* plants were Asp, Thr, Ser, Ala Lys, Gln, Arg, and Pro; of these, only Ser was lower in the leaves (Supplementary Table S2). The free amino acid content in the stem of the two variant OE lines was lower (**Figure 5A**) than in those of ZH11, and the amino acids that showed the higher reduction were Asp, Thr, Ser, Gly, Ala, Met, Ile, Leu, Tyr, Phe, His, Arg, and Pro (Supplementary Table S2). Nevertheless, the free amino acid content of OE1 and OE2 plants was relatively higher in the root and leaves than in those of ZH11, but lower in the stem (**Figure 5B**). Furthermore, we found that the free amino acid content was relatively higher in the roots of Ri and *osnpf7.7* plants than in those of ZH11, but lower in the leaves (**Figure 5B**). Nevertheless, the total nitrogen content of OE1 and OE2 lines was relatively higher in all organs than in those of ZH11, but lower in Ri and *osnpf7.7* plants (**Figure 5C**). Therefore, both splicing variants of *OsNPF7.7* participated in nitrogen allocation from the roots to the leaves and affected rice growth and development.

**FIGURE 6:**
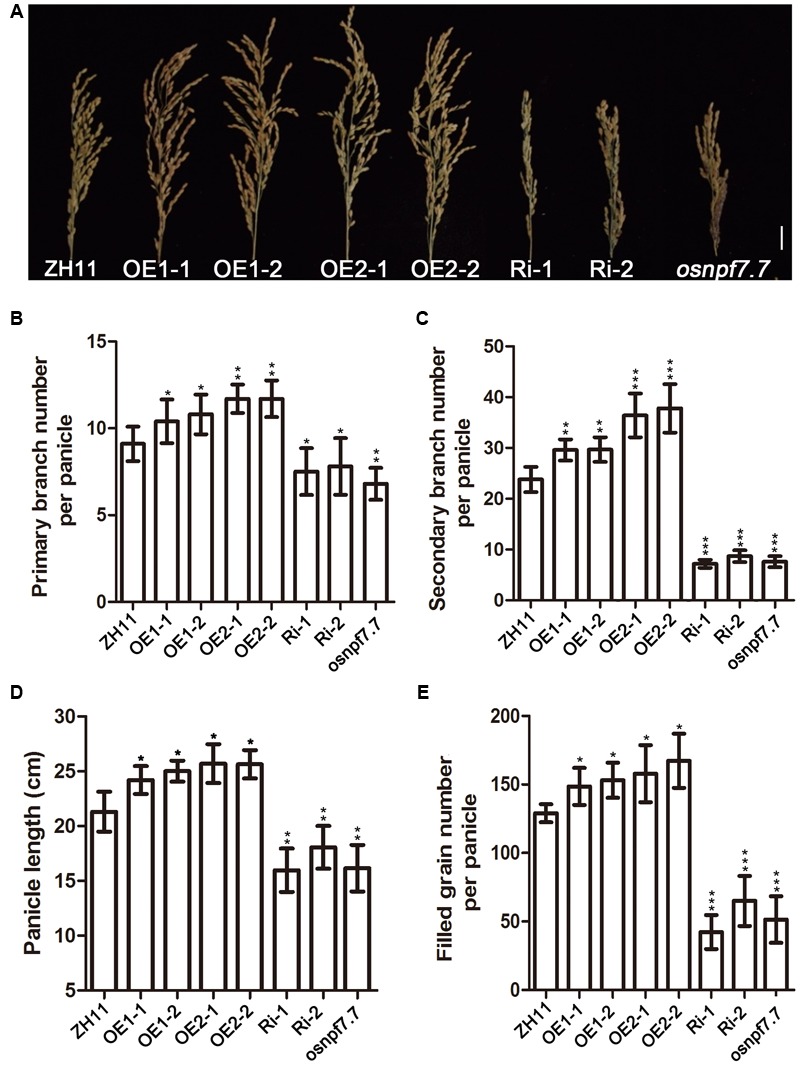
Phenotype of panicle at the mature stage of transgenic plants in ZH11. Phenotype of panicle at the mature stage **(A)**, primary branch number per panicle **(B)**, secondary branch number per panicle **(C)**, panicle length **(D)**, and filled grain number per panicle **(E)** of paddy field-grown ZH11 transgenic plants at mature stage. Three replicates have been used for each experiment. Significant levels (each transgenic line VS. ZH11): ^∗∗∗^*p* < 0.001, ^∗∗^*p* < 0.01, ^∗^*p* < 0.05. Error bars depict the SD (*n* = 30). Bar = 3 cm **(A)**.

The effects of altered *OsNPF7.7* expression on agronomic traits associated with the grain number were evaluated using paddy field-grown plants (**Figure 6A**). The primary branch number per panicle (**Figure 6B**), secondary branch number per panicle (**Figure 6C**), panicle length (**Figure 6D**), and filled grain number per panicle (**Figure 6E**) of OE2 lines were significantly greater than those of ZH11 and markedly higher than that in OE1 lines. Nevertheless, the same indices of Ri and *osnpf7.7* plants were significantly lower than those of ZH11 (**Figures 6B–E**). Therefore, we indicated that two splicing variants of *OsNPF7.7* also regulate the panicle branching in rice.

### Overexpression of Both Splicing Variants for *OsNPF7.7* Increases Grain Yield and Enhances Nitrogen Utilization Efficiency

**FIGURE 7:**
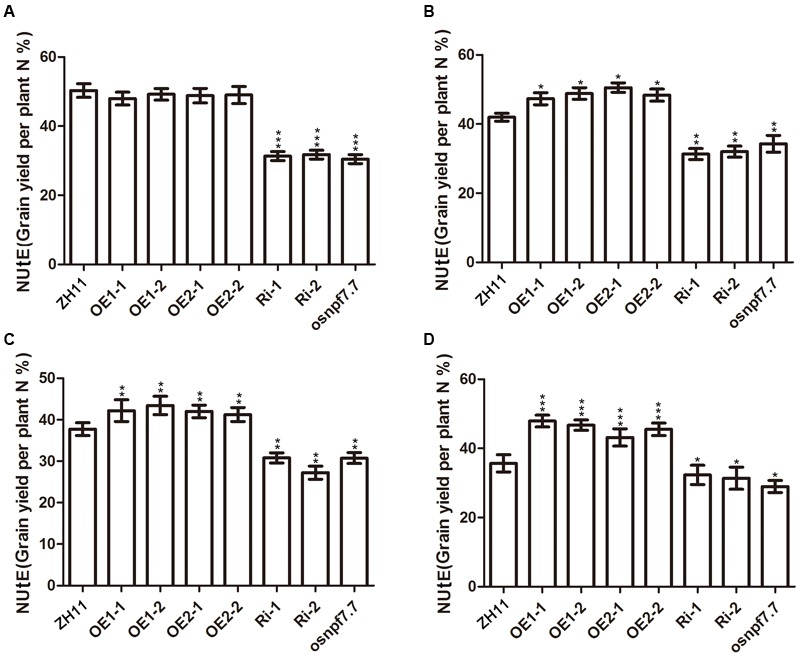
Nitrogen utilization efficiency under different nitrogen treatments of transgenic plants in ZH11. Nitrogen utilization efficiency at 0 kg hm^-2^ NH_4_NO_3_ **(A)**, 90 kg hm^-2^ NH_4_NO_3_ **(B)**, 180 kg hm^-2^ NH_4_NO_3_ **(C)**, and 270 kg hm^-2^ NH_4_NO_3_ **(D)**. The number of rice plants was 100 for each transgenic plants and ZH11. Three replicates have been used for each experiment. Significant levels (each transgenic line VS. ZH11): ^∗∗∗^*p* < 0.001, ^∗∗^*p* < 0.01, ^∗^*p* < 0.05. Error bars depict the SD (*n* = 3).

To further understand the potential practical application of *OsNPF7.7*, we evaluated the NUtE of transgenic plants in different concentrations of fertilizer under paddy field conditions. The results showed that NUtE of OE1 and OE2 lines was almost the same as that of ZH11, but the NUtE of Ri and *osnpf7.7* plants was lower in the no-nitrogen fertilizer treatment (**Figure 7A**). Overexpression of either *OsNPF7.7* variant led to better NUtE than that of ZH11 (**Figures 7B–D**). The NUtE of Ri and *osnpf7.7* plants decreased in all nitrogen treatments (**Figures 7B–D**).

Our study showed that *OsNPF7.7* played an important role in regulating tiller number and NUtE. Overexpression of both *OsNPF7.7-1* and *OsNPF7.7-2* in KY131, which is a *Japonica* rice variety with the largest planting area in China, greatly increased the tiller number (Supplementary Figures S2A–E) and the effective panicle number per plant (Supplementary Figures S2F–K). The effective panicle number per plant was higher than that in OE1 and OE2 lines, whereas that of KY131 was about 23 (Supplementary Figures S2F–K). Furthermore, OE1 and OE2 lines had a significantly higher filled grain number per plant than did KY131 plants (Supplementary Figure S2L). Therefore, both variants of *OsNPF7.7* enhanced rice grain yield by increasing the tiller number, effective panicle number, and filled grain number.

## Discussion

### Two Variants of *OsNPF7.7* Have Similar Expression Patterns Response to Nitrogen in Axillary Buds but Some Divergent Functions in Rice

Alternative splicing is one of patterns to increased functions of a gene at post transcriptional level (Kriventseva et al., 2003). In the present study, the newly found two variants of gene *OsNPF7.7* in the NPF family could be assigned by nitrogen sources, and the level of each variant depended on the concentration of external nitrogen source. The expression of *OsNPF7.7-1* and *OsNPF7.7-2* was downregulated in axillary buds at high external nitrogen concentration (**Figure 1D**). qRT-PCR also indicated that the gene was highly expressed in the axillary buds and panicle (**Figure 1F**). Therefore, we suggest that the gene function might be related to branching growth and development.

The long-splicing variant was localized in the plasma membrane, whereas the short-splicing variant in the vacuole membrane, probably because the former had the longest sequence at the N terminus, which determines the localization. It has been shown that some NPF family members are localized in the plasma membrane (Fang et al., 2013; Hu et al., 2015; Tang et al., 2017), and some are localized in the tonoplast membrane (Li et al., 2015; Hu et al., 2016). Therefore, the different localizations of two variants of *OsNPF7.7* suggest that each variant may functioned in different nitrogen transport process. In this study, our result indicated that overexpression of *OsNPF7.7-1* could increase nitrate influx and concentration, whereas overexpression of *OsNPF7.7-2* could enhance ammonium influx and concentration (Supplementary Figure S1). This demonstrated that *OsNPF7.7-1* and *OsNPF7.7-2* could balance the nitrate and ammonium uptake and storage at the plasma and vacuole membrane. Similarly, *OsNPF8.20-2* has been shown to positively influence the uptake of ammonium in rice, but not to directly transport ammonium (Fang et al., 2013).

### Enhanced Expression of Each Variants for *OsNPF7.7* Could Increase Rice Tiller Number by Promoting the Outgrowth of Axillary Bud

In rice, nitrogen nutrition can regulate root branching (López-Bucio et al., 2003; Walch-Liu et al., 2006). However, the regulation mechanism of tiller number via nitrogen fertilization remains unclear. The high-affinity transporter *OsNRT2.3* was a previously reported gene that has two variants, which differ in the regulation of rice growth and development. *OsNRT2.3a* is primarily expressed in root stellar cells (Tang et al., 2012), whereas *OsNRT2.3b* in the shoot phloem (Fan et al., 2016). Importantly, *OsNRT2.3b* regulates NUE (Fan et al., 2016). In addition, it was found that *OsNPF8.20-2* (Fang et al., 2013) and *OsNPF6.5* (Hu et al., 2015) regulate the tiller number, but the underlying mechanism was not clarified. In our study, we indicated that the two variants of *OsNPF7.7* controlled the tiller number (**Figure 3**) by regulating the axillary bud outgrowth (**Figure 4**). Previously, it has been reported that *OsGS1.2*, a key gene in nitrogen assimilation, was implicated in the regulation of axillary bud elongation (Ohashi et al., 2015, 2017). Our results showed that the expression of *OsGS1.2* was upregulated by the overexpression of *OsNPF7.7-2* and down-regulated by RNAi and *osnpf7.7* (**Figure 4**). Besides, overexpression of *OsNPF7.7-1* up-regulated the expression of the nitrate transporter *OsNPF6.5*, which regulates nitrate transport and tiller number in rice (Hu et al., 2015).

Additionally, the bud outgrowth marker *OsFC1* is required for axillary bud outgrowth and has been reported to decrease the tiller number (Minakuchi et al., 2010; Guo et al., 2013). And the strigolactone signaling gene *OsD3* also participates in the repression of the tiller number in rice (Jiang et al., 2013; Zhou et al., 2013). In the present study, we showed that overexpression of each variant for *OsNPF7.7* down-regulated the expression of *OsFC1* and *OsD3*, but RNAi and *osnpf7.7* up-regulated the expression of *OsFC1* and *OsD3*. Thus, both nitrogen and hormone processes might coordinate to regulate bud outgrowth, since previous study indicated that *OsGS1.2* increased the concentration of strigolactone (Yamaya and Kusano, 2014). Furthermore, the two kinds of overexpressing transgenic plants have almost the same phenotype of outgrowth bud. This is possible that enhanced expression of each variant could promote the elongation of the tiller buds by influencing the different nitrogen forms, since overexpression of *OsNPF7.7-1* promotes nitrate influx and concentration and overexpression of *OsNPF7.7-2* promotes ammonium influx and concentration (Supplementary Figure S1), and these two nitrogen forms are needed for plant growth and rice tillering (Fang et al., 2013; Hu et al., 2015; Tegeder and Masclaux-Daubresse, 2018).

### Both Variants of *OsNPF7.7* Have the Potential to Improve Grain Yield and Nitrogen Utilization Efficiency in Rice

In rice, the tiller number determines the potential effective panicle number (Yan et al., 1998; Xing and Zhang, 2010). Furthermore, it has been reported that the grain number per land area unit can be increased by increasing the panicle number or the grain number per panicle (Khush, 1995). In this study, we showed an increased effective panicle number and filled grain number accompanied by an increased tiller number in *OsNPF7.7-1* and *OsNPF7.7-2* overexpressing lines in KY131 (Supplementary Figure S2). Since the use of membrane transporters can improve crops and thus contribute to sustainable food production (Schroeder et al., 2013). Therefore, our results indicate that overexpression of each variant for *OsNPF7.7* could enhance rice grain yield by increasing of tiller number.

Transporters for nitrogen acquisition are essential for NUE (Tegeder, 2014). Although some efforts have been made in various plants, the improvement of NUE in crops is very limited (Li et al., 2017). In the present study, we found that enhanced expression of each variant for *OsNPF7.7* had better NUtE in the high-nitrogen and low-nitrogen treatments than that in ZH11, but no significant difference was found in the no-nitrogen treatment. These results suggest that overexpression of both variants for *OsNPF7.7* promotes and accelerates nitrogen translocation from the roots to the leaves and from the straws to the seeds, but had no effect on NUtE when nitrogen concentration in the environment was very low. It has been reported that overexpression of *OsNPF7.3* did not increase NUE in high ammonium supply (Fan et al., 2014). However, both variants of *OsNPF7.7* differed from *OsNPF7.3* with respect to the regulation of NUE, although both genes belong to the NPF subfamily. Overall, we showed that two variants of *OsNPF7.7* regulate shoot branching and NUtE in rice.

## Author Contributions

ZF designed the research. ZF, WH, GB, JW, WZ, QZ, and KL performed the experiments. ZF, WH, and SS drafted the manuscript.

## Conflict of Interest Statement

The authors declare that the research was conducted in the absence of any commercial or financial relationships that could be construed as a potential conflict of interest.

## Footnotes

1 http://rice.plantbiology.msu.edu/

2 http://rmd.ncpgr.cn/

3 http://www.cambia.org
