# Supplementary material for: Two Splicing Variants of OsNPF7.7 Regulate Shoot Branching and Nitrogen Utilization Efficiency in Rice
Source: Front Plant Sci. 2018 Mar 8;9:300. doi: 10.3389/fpls.2018.00300 (PMC5852072; doi:10.3389/fpls.2018.00300)
Supplement: Supplementary file 1 [file Table_1.DOCX]

Supplementary Material

Two splicing variants of *OsNPF7.7* regulate shoot branching and nitrogen utilization efficiency in rice

*Weiting Huang*^1+^, *Genxiang* *Bai*^1, 2+^, *Jie Wang*^1^, *Wei Zhu*^1^, Qisen Zeng^1, 2^, *Kai* *Lu*^1^, *Shiyong* *Sun*^2^ and *Zhongming* *Fang*^1, 2*^

^1^ *Center of Applied Biotechnology, Wuhan Institute of Bioengineering, Wuhan 430415, China*

^2^ *National Key Laboratory of Crop Genetic Improvement, Huazhong Agricultural University, Wuhan 430070, China*

***Corresponding authors**Zhongming Fang
zmfang@mail.hzau.edu.cn

^+^Weiting Huang and Genxiang Bai have contributed equally to this work.

# Supplementary Data

**Supplementary Table 1** List of the primers in this study

| **Name** | **Sequence (5' - 3')^a^** | **Note** |
| --- | --- | --- |
| ***OsNPF7.7 -GFP* fusion** | | |
| OE1-F | AGATCTATGGGCATGGAGAGCGGAGAT | For amplification of *OsNPF7.7-1* cDNA fragment to construct plasmid *pOsNPF7.7-1-GFP* |
| OE1-R | ACTAGTGTGCGAGTGCGGGACGGCGAG |  |
| OE2-F | AGATCTATGCCATATAAGTATGTACTA | For amplification of *OsNPF7.7-2* cDNA fragment to construct plasmid *pOsNPF7.7-2-GFP* |
| OE2-R | ACTAGTGTGCGAGTGCGGGACGGC |  |
| cDNA-*GFP*-DF | GATGTTGGCGACCTCGTATT | For detection of *OsNPF7.7-1* and *OsNPF7.7-2* cDNA-*GFP* transgenic plants. The length of fragment was 515 bp. |
| cDNA-*GFP*-DR | TCGTTATGTTTATCGGCACTTT |  |
| ***OsNPF7.7* overexpressing rice** | | |
| OE1-F | AGATCTATGGGCATGGAGAGCGGAGAT | For amplification *OsNPF7.7-1* cDNA fragment to construct plasmid *p35S*-*OsNPF7.7-1* |
| OE1-R | CTTAAGTCAGTGCGAGTGCGGGACGGCGAG |  |
| OE2-F | AGATCTATGCCATATAAGTATGTACTA | For amplification *OsNPF7.7-2* cDNA fragment to construct plasmid *p35S*-*OsNPF7.7-2* |
| OE2-R | CTTAAGTCAGTGCGAGTGCGGGACGGC |  |
| OE-DF | GATGTTGGCGACCTCGTATT | For detection of *OsNPF7.7-1* and *OsNPF7.7-2* over-expressing transgenic plants. The length of fragment was 515 bp. |
| OE-DR | TCGTTATGTTTATCGGCACTTT |  |
| ***OsNPF7.7* RNAi rice** | | |
| RNAi-1F | ACTAGTCATCCCATCACTACAACC | Amplification the reverse fragment of *OsNPF7.7* cDNA to construct plasmid *pOsNPF7.7i* |
| RNAi-1R | GAGCTCGTATCCAAACGACGACAG |  |
| RNAi-2F | GGTACCCATCCCATCACTACAACC | Amplification the forward fragment of *OsNPF7.7* cDNA to construct plasmid *pOsNPF7.7i* |
| RNAi-2R | GGATCCGTATCCAAACGACGACAG |  |
| RNAi-DF | GATGTTGGCGACCTCGTATT | For detection of *OsNPF7.7* RNAi transgenic plants. The length of fragment was 515 bp. |
| RNAi-DR | TCGTTATGTTTATCGGCACTTT |  |
| **qRT-PCR** | | |
| Actin1-q-F | CGGTGTCATGGTCGGAAT | For q-PCR of *OsActin1* (LOC_Os03g50885). |
| Actin1-q-R | GCTCGTTGTAGAAGGTGT |  |
| OsNPF7.7-Aq-F | GGAGATGCCCAACTGCCGCT | For q-quantitative and q-PCR of *OsNPF7.7-1* and *OsNPF7.7-2* |
| OsNPF7.7-Aq-R | CCAAGTTGAGGGAGATGCCAGCAT |  |
| OsNPF7.7-1q-F | TATATGTCCACAAACAAACAG | For q-quantitative and q-PCR of *OsNPF7.7-1* |
| OsNPF7.7-1q-R | GTTGTACTGGTCAGCTCCAAAG |  |
| OsNPF6.5q-F | TGGCGATGGTGTTGCCGGAGA | For q-quantitative and q-PCR of *OsNPF6.5* |
| OsNPF6.5q-R | AACAGCTCCGCCCCGAGGATCA |  |
| OsNRq-F | CCTGGAGAAGATGGGCTAT | For q-quantitative and q-PCR of *OsNR* |
| OsNRq-R | GCACAACCATCCATCAATC |  |
| OsAMT1.2q-F | ACGTGCTGCACAGGTTCGGGCT | For q-quantitative and q-PCR of *OsAMT1.2* |
| Os AMT1.2q-R | TCCGGCGACTTTCTGGGCTCCA |  |
| OsGS1.2q-F | TGTTTCTCCTCATCCCTGC | For q-quantitative and q-PCR of *OsGS1.2* |
| OsGS1.2q-R | TCACAGTCCTCGCTTTGC |  |
| OsFC1q-F | TCGTCCACCAATCTTGTGAGCACC | For q-quantitative and q-PCR of *OsFC1* |
| OsFC1q-R | GTTGGCGAACGCCATGATCACGTC |  |
| OsD3q-F | TCCAAACTTGCGGGACATGCAGTT | For q-quantitative and q-PCR of *OsD3* |
| OsD3 q-R | CCATTGCACAGTGGAGCAATGGCA |  |

**Supplementary Table 2** **Amino acid concentration (mg g^-1^ DW) of rice seedlings of 3-month-old different transgenic plant grown in paddy**

|  | ZH11 | OE1 | OE2 | Ri | *osnpf7.7* |
| --- | --- | --- | --- | --- | --- |
| Root |  |  |  |  |  |
| Asp | 1.917±0.412 | 1.804±0.438 | 1.869±0.083 | 3.933±0.177* | 3.112±0.232* |
| Thr | 2.348±0.443 | 1.307±0.316* | 2.422±0.697 | 4.271±0.221* | 4.153±0.829* |
| Ser | 4.364±0.503 | 5.432±0.276* | 4.536±0.078 | 5.816±0.431* | 7.350±0.796* |
| Glu | 2.984±0.204 | 2.679±0.244 | 3.012±0.065 | 5.143±0.384* | 3.180±0.189 |
| Gly | 4.367±0.233 | 3.561±0.527 | 2.920±0.075* | 3.566±0.161 | 5.000±0.591 |
| Ala | 8.079±0.588 | 5.836±0.183* | 5.609±0.047* | 9.467±0.700* | 9.855±0.635* |
| Cys | 1.521±0.176 | 1.506±0.016 | 2.430±0.025* | 2.582±0.098* | 1.741±0.199 |
| Val | 2.434±0.562 | 3.063±0.389 | 3.058±0.055 | 3.674±0.594 | 1.958±0.187 |
| Met | 1.261±0.216 | 1.868±0.014* | 1.868±0.014* | 2.205±0.057* | 1.521±0.065 |
| Ile | 0.408±0.072 | 0.394±0.092 | 0.469±0.004 | 0.593±0.150 | 0.483±0.058 |
| Leu | 1.280±0.175 | 1.072±0.277 | 1.320±0.005 | 1.723±0.473 | 1.414±0.138 |
| Tyr | 1.997±0.234 | 2.558±0.405* | 3.810±0.041* | 3.676±0.307* | 1.805±0.141 |
| Phe | 0.988±0.192 | 1.727±0.265* | 1.787±0.021* | 2.083±0.365* | 0.944±0.071 |
| Lys | 0.848±0.053 | 1.364±0.080 | 1.268±0.019 | 1.755±0.203* | 1.520±0.212* |
| Gln | 3.921±0.347 | 6.122±0.575* | 7.205±0.225* | 6.775±0.675* | 7.969±0.553* |
| His | 0.530±0.196 | 0.574±0.064 | 0.579±0.031 | 0.634±0.128 | 0.543±0.015 |
| Arg | 0.792±0.060 | 0.629±0.082 | 0.606±0.015 | 0.900±0.292* | 0.956±0.042* |
| Pro | 1.136±0.142 | 1.004±0.245 | 0.995±0.027 | 1.679±0.392 | 1.644±0.165* |
| Total | 41.276±4.758 | 42.499±3.082 | 43.897±3.689 | 60.077±2.972* | 55.149±3.549* |
| Leaf Sheath |  |  |  |  |  |
| Asp | 4.962±0.295 | 2.495±0.567* | 1.346±0.192* | 3.615±0.756* | 2.413±0.748* |
| Thr | 14.249±0.294 | 8.794±0.464* | 5.287±0.262* | 13.229±0.691 | 10.888±1.506* |
| Ser | 15.152±2.284 | 9.622±0.633* | 6.000±0.115* | 11.369±1.151* | 12.353±1.034 |
| Glu | 6.710±0.872 | 3.870±0.717* | 6.916±0.271 | 7.700±0.473 | 3.649±0.476* |
| Gly | 3.991±0.722 | 2.364±0.505* | 2.083±0.075* | 3.901±0.710 | 4.681±0.543 |
| Ala | 18.583±2.495 | 9.891±0.732* | 9.085±0.209* | 21.435±1.402 | 17.920±1.643 |
| Cys | 1.635±0.291 | 1.689±0.203 | 2.260±0.034 | 2.146±0.040 | 1.543±0.243 |
| Val | 3.888±0.415 | 3.111±0.295 | 3.323±0.049 | 2.647±0.302* | 3.217±0.417 |
| Met | 6.064±0.576 | 3.698±0.484* | 4.529±0.145* | 4.422±0.804* | 6.303±0.600 |
| Ile | 0.860±0.150 | 0.563±0.117* | 0.590±0.008* | 0.455±0.098* | 0.818±0.223 |
| Leu | 3.564±0.605 | 2.137±0.454* | 2.312±0.032* | 2.004±0.454* | 3.606±0.810 |
| Tyr | 6.270±0.522 | 4.463±0.389* | 5.385±0.093* | 5.801±0.121 | 4.406±0.412* |
| Phe | 4.605±0.647 | 3.085±0.485* | 3.680±0.033* | 2.762±0.447* | 3.538±0.411* |
| Lys | 1.565±0.259 | 1.260±0.260 | 1.379±0.030 | 1.024±0.201 | 1.433±0.518 |
| Gln | 7.513±0.687 | 7.219±0.689 | 6.842±0.430 | 9.163±0.407* | 9.332±1.154* |
| His | 1.066±0.234 | 0.788±0.169* | 0.676±0.085* | 0.745±0.117 | 0.863±0.220 |
| Arg | 2.564±0.443 | 2.022±0.073* | 1.640±.026* | 1.467±0.349* | 2.124±0.568 |
| Pro | 8.009±0.054 | 4.005±0.541* | 7.577±0.256 | 10.992±0.472* | 5.929±0.729* |
| Total | 111.255±10.381 | 71.071±4.478 | 70.912±5.0364 | 104.882±7.366 | 95.017±6.919 |
| Leaf |  |  |  |  |  |
| Asp | 1.747±0.040 | 4.710±0.585* | 3.394±0.102* | 1.137±0.222 | 1.590±0.427 |
| Thr | 4.282±0.132 | 11.061±1.800* | 8.541±0.510* | 3.295±0.122 | 3.314±0.574 |
| Ser | 4.552±0.108 | 12.831±1.190* | 2.653±0.067* | 3.506±0.565* | 3.658±1.117* |
| Glu | 11.962±0.236 | 5.397±0.758* | 9.263±0.893* | 12.231±0.259 | 10.833±0.460 |
| Gly | 4.356±0.090 | 3.140±0.424* | 3.029±0.185* | 3.763±0.669 | 3.558±1.074* |
| Ala | 19.039±0.412 | 13.683±0.509* | 13.483±0.699* | 18.548±0.604 | 19.637±0.633 |
| Cys | 2.243±0.020 | 1.872±0.027 | 2.074±0.078 | 2.228±0.055 | 1.577±0.154* |
| Val | 4.595±0.114 | 3.928±0.645 | 3.515±0.567 | 3.811±0.467 | 4.452±0.879 |
| Met | 7.887±0.188 | 4.859±0.487* | 6.408±0.128* | 7.297±0.894 | 6.924±0.508* |
| Ile | 1.319±0.034 | 0.784±0.188* | 1.026±0.038 | 1.074±0.151 | 1.441±0.313 |
| Leu | 3.214±0.085 | 3.063±0.789 | 2.543±0.084* | 2.706±0.393* | 2.641±0.665 |
| Tyr | 5.524±0.065 | 5.351±0.815 | 4.848±0.590 | 5.610±0.370 | 3.640±0.675* |
| Phe | 4.382±0.080 | 4.023±0.812 | 4.063±0.079 | 3.743±0.456* | 3.421±0.582* |
| Lys | 1.276±0.020 | 1.719±0.457* | 0.633±0.069* | 0.958±0.134 | 1.795±0.461 |
| Gln | 5.841±0.189 | 8.803±0.636* | 6.129±0.384* | 5.893±0.612 | 5.559±0.678 |
| His | 1.032±0.035 | 1.144±0.388 | 1.643±0.006* | 0.743±0.142* | 1.129±0.163 |
| Arg | 1.740±0.065 | 2.451±0.323* | 1.476±0.030 | 1.291±0.209 | 1.809±0.335 |
| Pro | 1.102±0.030 | 8.141±0.889* | 2.311±0.051* | 1.624±0.294 | 1.080±0.385 |
| Total | 86.091±5.681 | 96.960±6.017* | 78.035±5.484* | 79.460±5.752* | 78.060±5.080* |

“*” indicated significant differences (Each transgenic plants VS ZH11) at P < 0.05. Date are shown as mean ± SD (n =4). Three replicates have been used for each experiment.


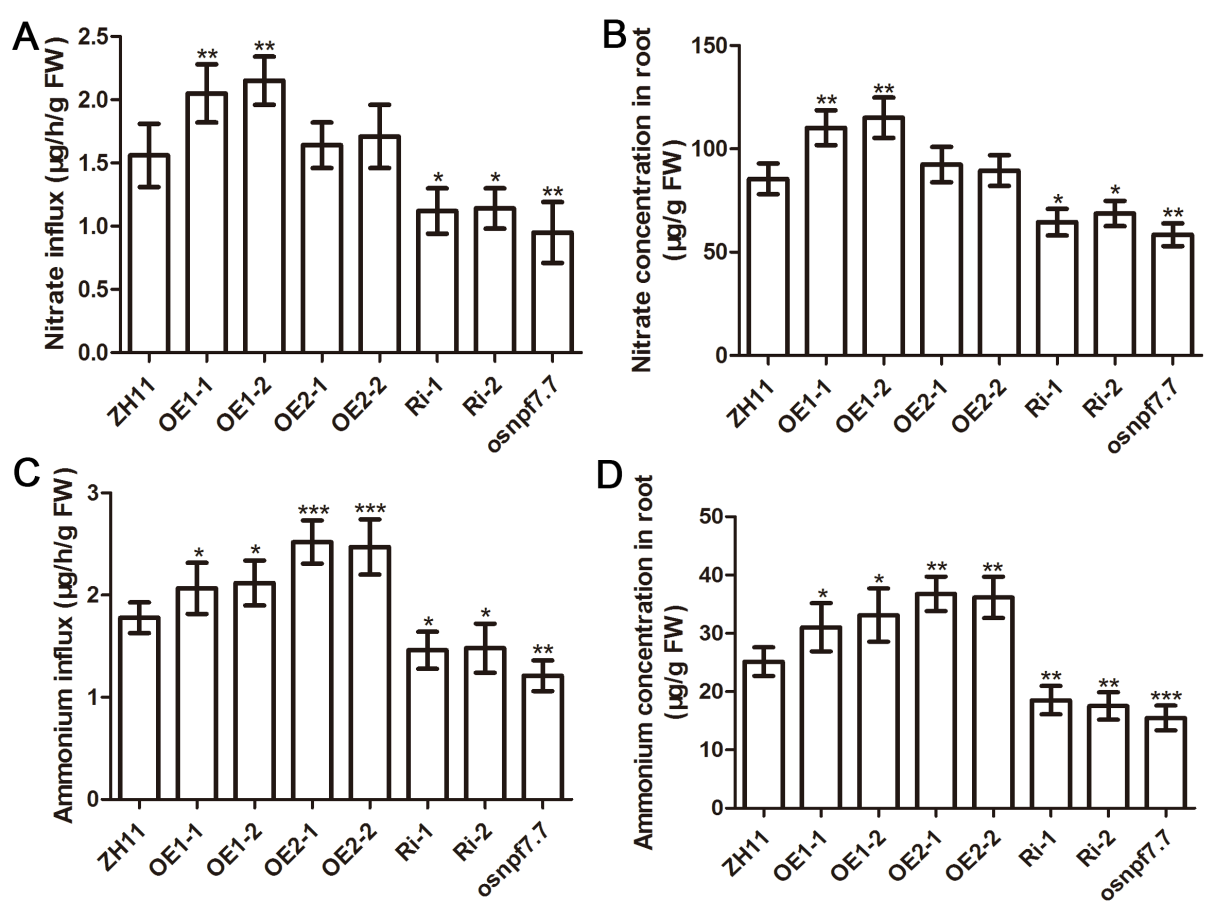


**Supplementary figure 1 *OsNPF7.7* influenced** **NO_3_^-^ influx, NO_3_^-^ concentration, NH_4_^+^ influx, and NH_4_^+^ concentration among transgenic lines.** (A) Analysis of NO_3_^-^ influx rate among ZH11, OE1 lines, OE2 lines, Ri lines and *osnpf7.7* cultured under 2.0 mM NaNO_3_ supply. (B) NO_3_^-^ concentration of ZH11, OE1 lines, OE2 lines, Ri lines and *osnpf7.7* cultured under 2.0 mM NaNO_3_ supply. (C) Analysis of NH_4_^+^ influx rate among ZH11, OE1 lines, OE2 lines, Ri lines and *osnpf7.7* cultured under 1.0 mM (NH_4_)_2_SO_4_ supply. (D) NH_4_^+^ concentration of ZH11, OE1 lines, OE2 lines, Ri lines and *osnpf7.7* cultured under 1.0 mM (NH_4_)_2_SO_4_ supply. Three replicates have been used for each experiment. “*”, “**” and “***” indicated significant differences (Each transgenic line VS ZH11) at P < 0.05, P < 0.01 and P < 0.001, respectively. Date are shown as mean ± SD (n =10).


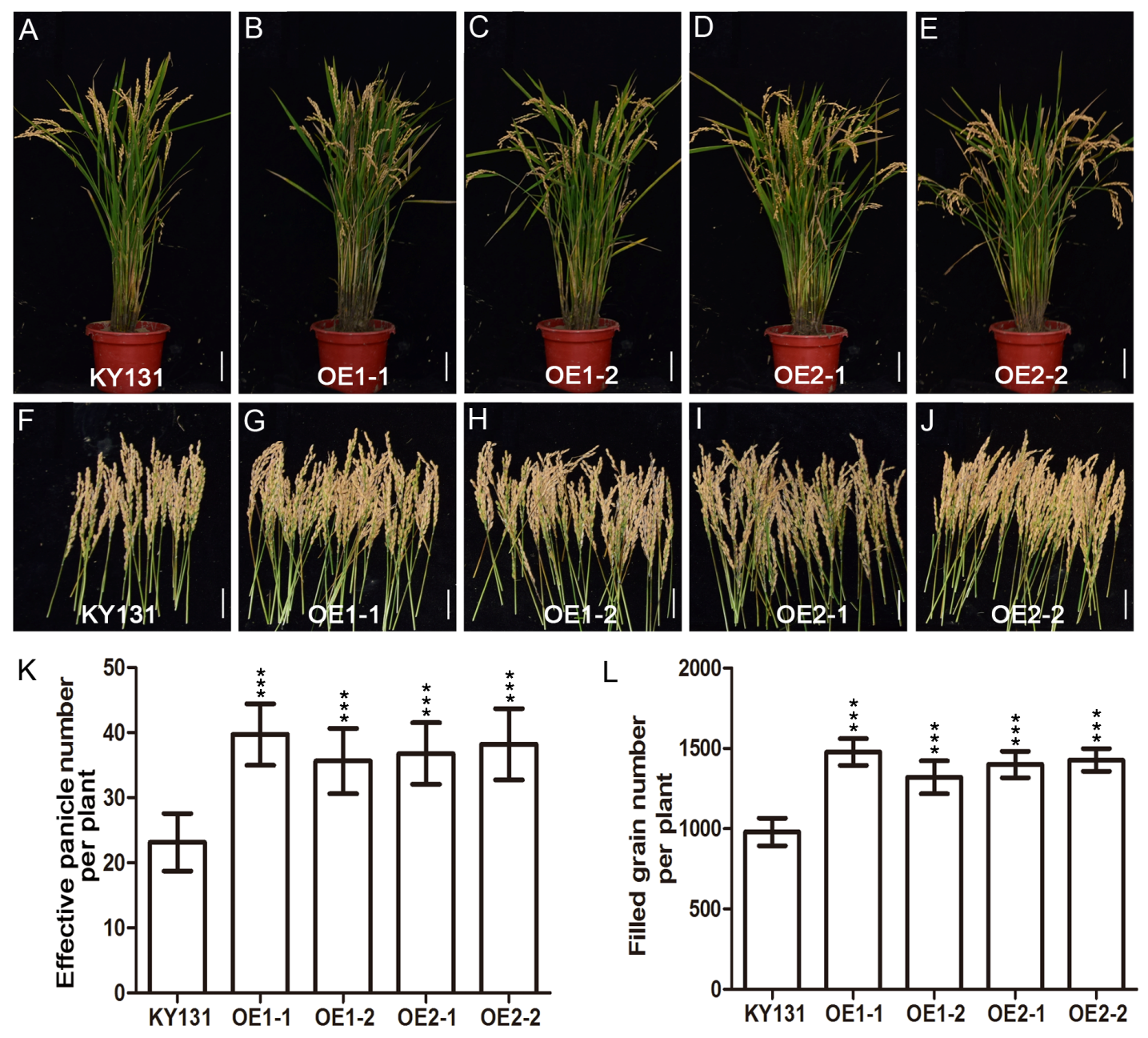


**Supplementary figure 2 Phenotype of paddy field-grown *OsNPF7.7* transgenic plants in KY131**

KY131 plants (A), *OsNPF7.7-1*-overexpressing lines (OE1-1 and OE1-2; B, C), and *OsNPF7.7-2*-overexpressing lines (OE2-1 and OE2-2; D, E) under paddy field conditions. Effective panicle number of KY131 (F), OE1-1 (G), OE1-2 (H), OE2-1 (I), and OE2-2 (J) was showed. Effective panicle number (K) and filled grain number per plant (L) was calculated. Three replicates have been used for each experiment. “*”, “**” and “***” indicated significant differences (Each transgenic line VS KY131) at P < 0.05, P < 0.01 and P < 0.001, respectively. Date are shown as mean ± SD (n=30). Bars = 10 cm (A-E).
